# *RHRVEasy*: Heart rate variability made easy

**DOI:** 10.1371/journal.pone.0309055

**Published:** 2024-11-27

**Authors:** Constantino A. García, Sofía Bardají, Pablo Pérez-Tirador, Abraham Otero

**Affiliations:** Department of Information Technology, Escuela Politécnica Superior, Universidad San Pablo-CEU, CEU Universities, Campus Montepríncipe, Boadilla del Monte, Madrid, Spain; Institute for Basic Science, KOREA, REPUBLIC OF

## Abstract

Heart Rate Variability (HRV) analysis aims to characterize the physiological state affecting heart rate, and identify potential markers of underlying pathologies. This typically involves calculating various HRV indices for each recording of two or more populations. Then, statistical tests are used to find differences. The normality of the indices, the number of groups being compared, and the correction of the significance level should be considered in this step. Especially for large studies, this process is tedious and error-prone. This paper presents *RHRVEasy*, an R open-source package that automates all the steps of HRV analysis. *RHRVEasy* takes as input a list of folders, each containing all the recordings of the same population. The package loads and preprocesses heart rate data, and computes up to 31 HRV time, frequency, and non-linear indices. Notably, it automates the computation of non-linear indices, which typically demands manual intervention. It then conducts hypothesis tests to find differences between the populations, adjusting significance levels if necessary. It also performs a post-hoc analysis to identify the differing groups if there are more than two populations. *RHRVEasy* was validated using a database of healthy subjects, and another of congestive heart failure patients. Significant differences in many HRV indices are expected between these groups. Two additional groups were constructed by random sampling of the original databases. Each of these groups should present no statistically significant differences with the group from which it was sampled, and it should present differences with the other two groups. All tests produced the expected results, demonstrating the software’s capability in simplifying HRV analysis. Code is available on https://github.com/constantino-garcia/RHRVEasy.

## 1 Introduction

Heart rate variability (HRV) refers to continuous variations in the distances between consecutive beats [1, 2]. Heart beats originate at the sinus node, whose activity is modulated by parasympathetic and sympathetic nerves. Parasympathetic nerves reduce the frequency of the electrical impulse generation, and hence decrease heart rate (HR), while sympathetic nerves increase both the frequency of the electrical impulse and HR. Additionally, systems like the Renin Angiotensin Aldosterone System (RAAS) and Respiratory Sinus Arrhythmia (RSA) continuously modulate HR in response to various stimuli [3, 4].

**Fig 1:**
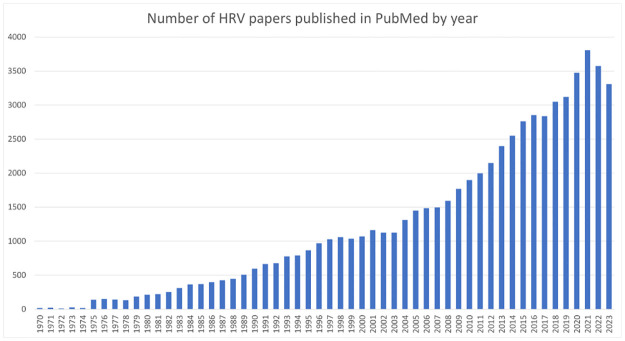
HRV articles per year. Number of articles related to heart rate variability analysis published in PubMed per year.

The term Heart Rate Variability Analysis (HRVA) encompasses techniques that study the variations between interbeat distances (the so-called RR intervals) to obtain information about the different physiological systems influencing HR. Since the seminal study by Hon and Lee in 1963 [5], tens of thousands of scientific publications on potential clinical applications of HRV have been published [6–13], and thousands of new articles continue to appear each year (see Fig 1).

In typical HRV studies, RR recordings from multiple populations undergo HRV index calculation using various tools such as Kubios HRV [14], gHRV [15], the HRV Toolkit of the WFDB Toolbox [16] or *RHRV* [17]. These indices are typically classified into three main categories: time-domain, frequency-domain, and non-linear indices [2]. This process, especially in large studies, can be laborious and repetitive, particularly with graphical tools lacking automation.

Then, these indices are imported into a statistical tool for population comparison. Some HRV toolkits can generate up to 30–40 different indices per recording [14, 17]. Therefore, in addition to assessing the normality of the indices and the number of groups to be compared, the correction of the level of significance of the statistical tests should be considered. Again, this process can be complex and error-prone, and may involve repetitive manual tasks.

We have created a tool to automate a complete HRVA. We have built this tool on top of the *RHRV* package [17], an open-source R package born in 2008 to carry out HRV analysis, which currently has about 1,000 monthly downloads according to the Comprehensive R Archive Network (CRAN) statistics. The tool, named *RHRVEasy*, takes as inputs the recordings of all the experimental groups of the study and it calculates up to 31 time, frequency, and non-linear domain HRV indices. The tool then streamlines statistical analysis by automating decisions related to data normality, the necessity for significance level corrections, and the post-hoc analysis if the study involves more than two populations.

## 2 Materials and methods

**Fig 2:**
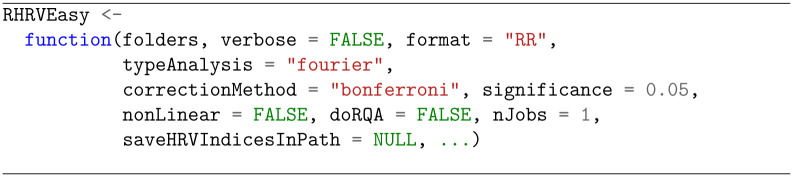
Prototype of the main RHRVEasy function.

*RHRVEasy* enables the user to carry out a full HRVA by just invoking a function with a single mandatory parameter: a list with the folders containing the recordings of the experimental groups (see Fig 2). The tool automatically assigns the name of each experimental group based on the folder in which its recordings are stored.

The remaining function parameters have default values that can be overwritten by the user if desired. A verbose mode can be activated by setting verbose to TRUE. The format parameter specifies the format in which the RR intervals are stored. All formats supported by the *RHRV* package can be used: WFDB, ASCII, RR, Polar, Suunto, EDFPlus, or Ambit [17]. The default format is RR, where the beat distances in seconds are stored in a single column of an ASCII file. The rest of the parameters of the function RHRVEasy shall be explained when their usage is introduced.

**Fig 3:**
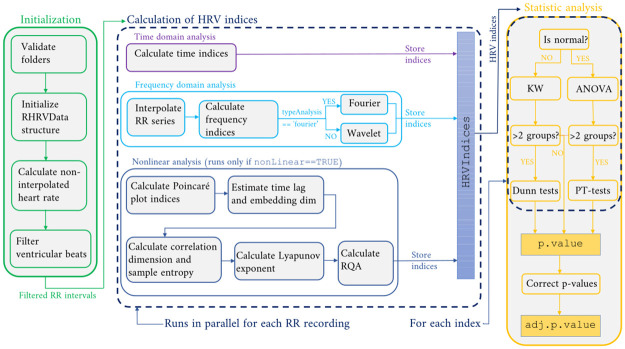
*RHRVEasy* fluxogram. Flowchart with the operations performed by the *RHRVEasy* package. Rectangles with rounded edges represent operations and rectangles with square borders represent data.frames or lists. In this figure, KW refers to Kruskal-Wallis and PT-test to Paired T-test.

After validating the folders parameter (see Fig 3), RHRVEasy loads the RR intervals from each recording into an *RHRV* data structure named RHRVData. Then, it constructs a time series from the distances between beats, representing the non-interpolated HR.

The final step of the initialization process involves filtering artifacts, missing detections, and ventricular beats. Both artifacts and missing detections are due to errors in the beat detector, usually caused by poor signal quality. Artifacts occur when the beat detector mistakenly identifies an artifact (typically high-frequency noise) as a beat, while missing detections occur when real beats are not detected. False detections lead to shorter-than-usual RR intervals, while missing detections result in longer-than-usual RR intervals. Ventricular beats, on the other hand, are disregarded due to their origin from myocardial automatism, which is not influenced by the autonomic nervous system and thus they are unsuitable for HRV analysis. The filtering algorithm employs adaptive thresholds to reject values inconsistent with typical physiological ranges [17].

This automatic filtering procedure aligns with the aim of *RHRVEasy* to fully automate HRV analysis. However, in some circumstances, it may be desirable to manually filter the RR intervals. This can be done using the EditNIHR function from *RHRV* [17], which launches a graphical editor that permits the user to manually eliminate outliers and atypical intervals. The edited file can then be saved and included in the folders containing the recordings of the experimental groups.

### 2.1 HRV indices computation

This section details the computation of HRV indices using time-domain, frequency-domain, or non-linear analysis techniques. All computations use sensible default parameters that may be overwritten using the special argument … (see Fig 2). S1 Table lists all time and frequency-domain indices that can be computed with *RHRVEasy*, while S2 Table lists the non-linear HRV indices. Both tables provide a brief description of the indices and the default parameters used for the calculations.

**Fig 4:**
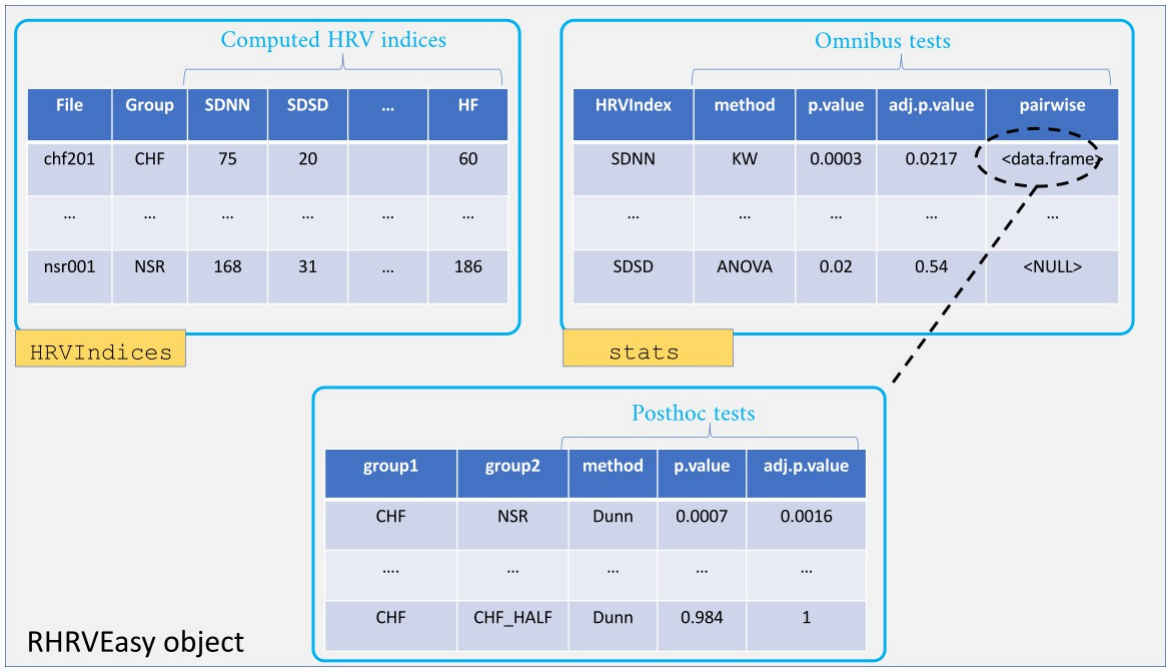
*RHRVEasy* results object. Structure of the object returned by the function RHRVEasy. Each table is a data.frame, with its name shown highlighted.

All the resulting indices are combined into a single data.frame named HRVIndices (see Fig 4), which can be saved to an Excel spreadsheet by specifying the saveHRVIndicesInPath argument in the RHRVEasy call (see Fig 2).

#### 2.1.1 Time and frequency-domain indices calculation

For each recording within each experimental group, several temporal HRV indices are computed, including SDNN, SDANN, SDNNIDX, pNN50, SDSD, rMSSD, IRRR, MADRR, TINN, and HRVi [1, 2]. The width of the sliding window used for the computations of SDANN and SDNNIDX is set to 300 seconds by default, as recommended by [1]. However, different values may be used, especially in animal experimentation, with values as low as 60 seconds [18] or even 10 seconds [19].

Due to the use of this sliding window, calculations for SDANN and SDNNIDX may fail. If a recording is short and comprises only one window, or if it contains at least one window of missing data, SDANN and SDNNIDX cannot be computed. In such instances, these indices are set to NA (Not Available), a warning message is generated, and these indices are ignored in the subsequent statistical analysis. However, these *NA* values may be reviewed and corrected manually later. After manual review and computation of any missing indices, the user can update the HRVIndices slot of the RHRVEasy object (see Fig 4) and rerun the statistical analysis using the RHRVEasyStats function provided for this purpose.

Regarding the frequency-domain analysis, the typeAnalysis parameter (see Fig 2) allows for choosing between the Fourier transform or the Wavelet transform to estimate spectral power across predefined frequency bands, including Ultra Low Frequency (ULF, typically below 0.03 Hz), Very Low Frequency (VLF, 0.03–0.05 Hz), Low Frequency (LF, 0.05–0.15 Hz), and High Frequency (HF, 0.15–0.4 Hz). These default spectral bands align with those commonly used in studies involving adult humans under resting conditions.

Since the RR interval series is not equally spaced [1], interpolation is necessary before applying any frequency analysis. Linear interpolation is used by default, interpolating at 4 Hz [20]. In the default configuration, a Fourier-based method is used. This method first removes any linear trend from the time series, then computes the periodogram and smooths it using modified Daniell kernels [21, p. 157].

If the typeAnalysis is set to “wavelet” the algorithm presented in [22] is used to estimate the instantaneous spectral power, which represents the distribution of power across frequency bands at specific time points. Then, the instantaneous spectral power is averaged over time to estimate the spectral power.

#### 2.1.2 Non-linear indices calculation

The calculation of certain non-linear HRV indices, particularly Recurrence Quantification Analysis (RQA) [23, 24], can be computationally intensive. This is why the RHRVEasy function defaults the boolean parameters nonLinear and doRQA to FALSE. Only if these parameters are explicitly changed will the non-linear analysis and RQA be executed; otherwise, only time and frequency indices will be computed.

The first non-linear indices are derived from the Poincaré plot, a geometric technique plotting each RR against the next one [25]. This method involves fitting an ellipse to the data and calculating the minor axis (*SD*_1_ index) and the major axis (*SD*_2_ index). *SD*_1_ characterizes short-term variability, while *SD*_2_ characterizes long-term variability.

The computation of the remaining non-linear indices initiates with the estimation of the time lag and embedding dimension, which are necessary for reconstructing the phase space according to the embedding theorem [26]. To determine the time lag, the autocorrelation function of the RR time series is computed. The first minimum is sought, and if absent, the first point decreasing to 1/*e* of the maximum is identified. If these criteria aren’t met, the average mutual information is employed with the same criteria [27]. If all these attempts fail, the time lag is set to a heuristic value of 30, determined through experimentation to sufficiently minimize autocorrelation. After the time lag has been estimated, the embedding dimension is estimated using the algorithm of Cao [28] (referred to as embeddingDimension hereafter).

**Fig 5:**
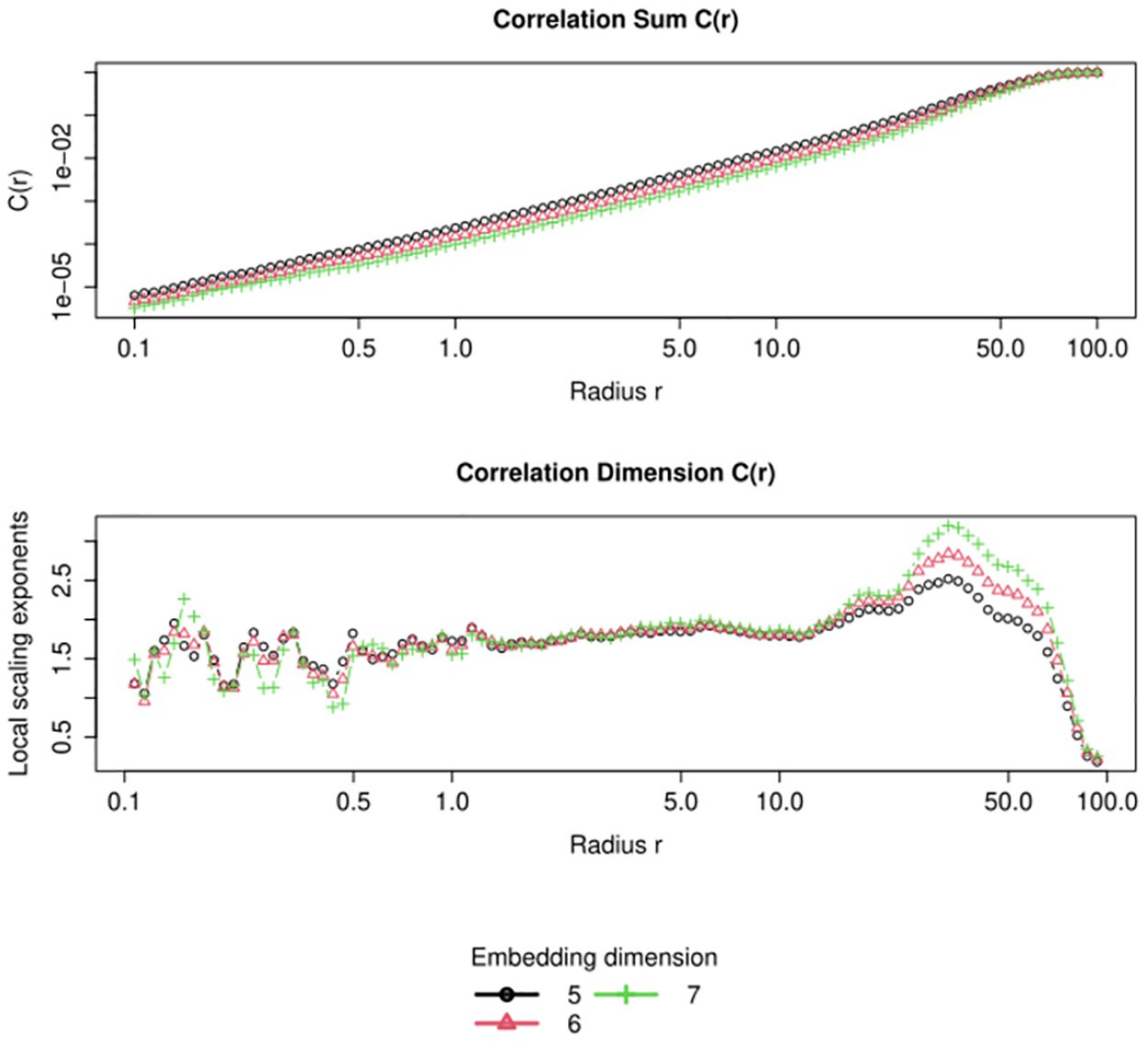
Plots used to estimate the correlation dimension. The upper plot shows the correlation sum versus the log of the radius, and the lower plot the local scaling exponent versus the log of the radius. The scaling region corresponds to radii between approximately 2 and 15.

The correlation dimension, the first non-linear index from phase space reconstruction, is calculated by first plotting the correlation sum correlation sum *C*(*m*, *r*) against the radius *r* for various embedding dimensions *m* [29] (see Fig 5). The data analyst identifies a linear region, known as the *scaling region*, where the slope of the correlation sum remains constant across all embedding dimensions. To find this region, the local slopes of the correlation sum with respect to changes in radius (the so-called local scaling exponent) are also plotted in log-log scale. The scaling region is preceded by an oscillatory region (radius < 0.7 in Fig 5) and a *noise regime* (0.7 < radius < 2), and followed by a *macroscopic regime* (radius > 15). The correlation dimension is determined by the slope of the correlation sum within the scaling region (radius between 2 and 15 in Fig 5).

Automating the identification of these regions, which is typically done visually, is crucial for *RHRVEasy*. To achieve this, a piecewise linear regression with four different regions is fitted to the plot of the local scaling exponents. The flattest slope region, representing the scaling region, is used to estimate the correlation dimension. The sample entropy [30] is calculated using this same region.

Both the correlation dimension and sample entropy are system invariants, meaning their estimates should be independent of the embedding dimension if this is sufficiently large [29]. Therefore, the final values of these indices are obtained by averaging three estimates, each computed with dimensions of the reconstructed phase space within the interval [embeddingDimension, embeddingDimension + 2].

The estimation of the Lyapunov exponent [31, 32] employs a strategy similar to that of the correlation dimension. However, it utilizes two regions for piecewise regression, as this corresponds to the expected number of regions in a Lyapunov plot.

Note that the computation of the Lyapunov exponent is based on the concept of *close trajectories*. This concept is implemented by examining trajectories within a small radius, where the specific value of *small* depends on the recording. To find a reasonable small radius, *r*_*small*_, we looked for the average correlation dimension verifying $\bar{C}\left(r_{small}\right)=10^{-3}$. This heuristic is based on the interpretation of $\bar{C}\left(r\right)$, which represents the average probability of finding a neighbor in the phase space within a radius *r*.

Finally, a RQA is also performed [23, 24]. RQA quantifies the number and duration of the recurrences, i.e., the number of times a trajectory in the phase space returns to a neighborhood of a region that was visited before. The same radius (*r*_*small*_) utilized in the Lyapunov calculations is employed for determining the neighborhood for RQA.

Except for Poincaré plot indices, computing other non-linear indices may fail. This could be due to convergence issues in the piecewise linear regressions or divisions by zero in RQA. In such cases, the statistic’s value is set to *NA* and excluded from the statistical analysis. Similarly to time and frequency-domain indices (see Section 2.1.1), these *NA* values may be manually reviewed and corrected later.

### 2.2 Parallelization of the HRV indices calculations

The calculation of HRV indices, particularly non-linear ones, can be time-consuming. To optimize computational resources, we implemented parallelization using the R package *foreach*. This parallelization operates on a per-recording basis, enabling simultaneous processing of multiple recordings and significantly reducing analysis time. The degree of parallelization is controlled by the nJobs parameter in the RHRVEasy function, which defaults to using a single core.

### 2.3 Statistical analysis

After calculating the HRV indices, the following step is the statistical analysis. Initially, an ANOVA model is constructed, followed by an evaluation of the normality of residuals using the Shapiro-Wilk test. In instances where the test yields significance, indicating non-conformity to a Gaussian distribution, the ANOVA model is discarded, and a Kruskal-Wallis test is employed.

The default significance level is 0.05, adjustable via the significance parameter. Due to multiple tests on HRV indices, a significance level correction is necessary. The default method is Bonferroni [33], known for its conservatism, which can reduce statistical power and increase Type II errors. Other correction methods are available through the correctionMethod parameter: family-wise error rate control methods (“bonferroni”, “holm”, “hochberg” and “hommel”) minimize Type I errors, while False Discovery Rate (FDR) control methods (“BH” [Benjamini & Hochberg], “fdr” [FDR] and “BY” [Benjamini & Yekutieli]) offer higher power at the expense of increased Type I errors. The “none” option does not correct p-values.

If statistically significant differences are found for an index and there are three or more groups, there needs not be statistically significant differences between all pairs of groups. In such a scenario, post-hoc tests permit identifying which pairs of groups present differences. Depending on whether ANOVA or Kruskal-Wallis serves as the omnibus test, Paired T-tests or Dunn post-hoc [34] are employed, respectively. Again, a correction to the significance level may also be applied to adjust the multiple post-hoc tests.

**Fig 6:**
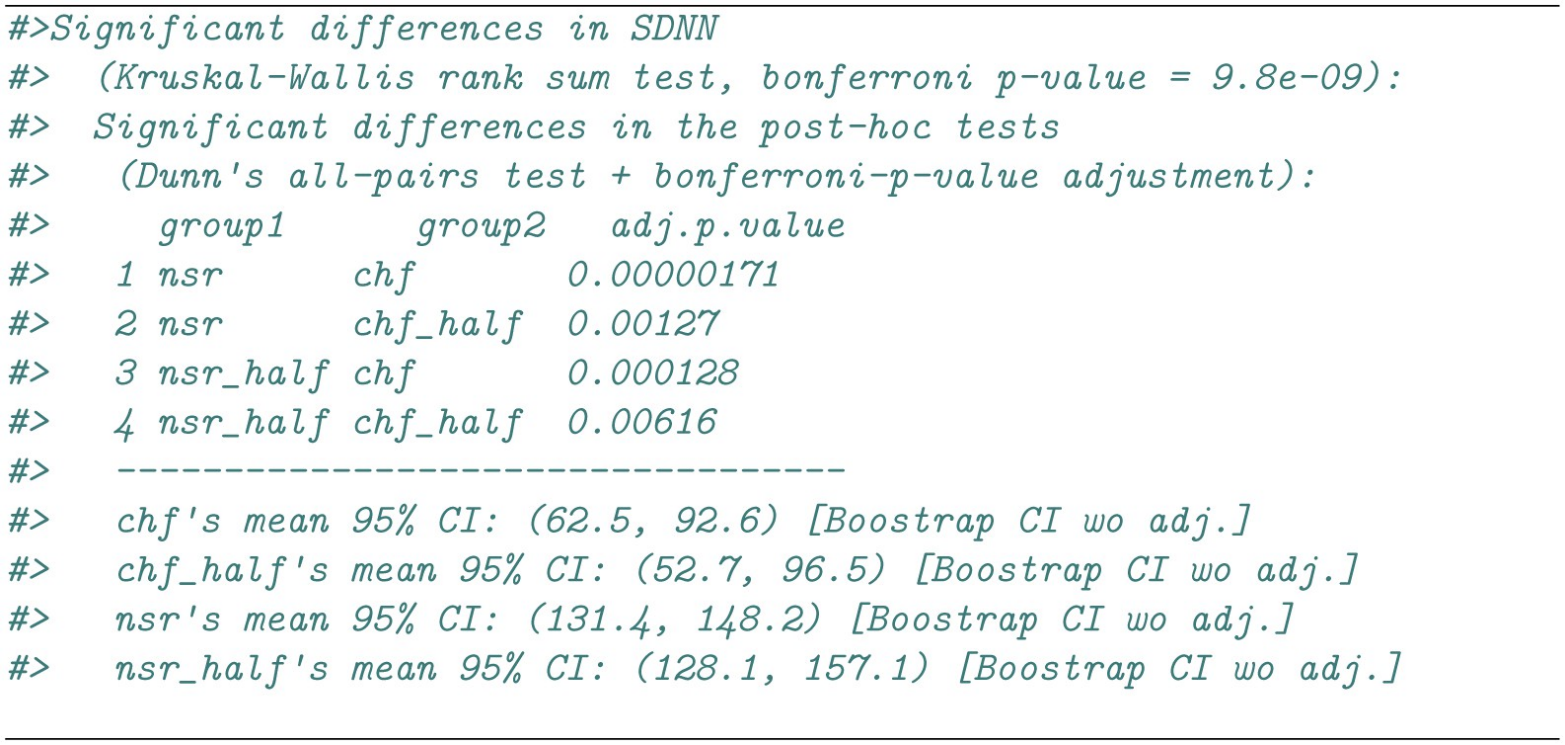
Example of RHRVEasy object output when displayed on the console (fragment). The output in this listing is adapted for paper width constraints.

The complete results of the statistical tests for each HRV index (ANOVA+paired T-Tests or Kruskal-Wallis+Dunn tests) are available as a data.frame under the slot stats of the RHRVEasy object, including the corrected and uncorrected p-values (see Figs 3 and 4). Furthermore, when displayed in the console, the RHRVEasy object provides a summary of the indices with statistically significant values, including p-values and unadjusted confidence intervals. The computation of these intervals involves T-test-based calculations for normal distributions and Bootstrap techniques otherwise [35]. Fig 6 shows the output of a RHRVEasy object.

## 3 Results

The *RHRVEasy* package was tested using recordings from two publicly available databases: the Normal Sinus Rhythm (NSR) RR Interval Database [36] for healthy subjects, and the Congestive Heart Failure (CHF) RR Interval Database [36] for patients with congestive heart failure. Further details about these databases can be found in S1 Appendix.

To validate the package’s performance when working with more than two experimental groups, about half of the recordings from both databases were randomly selected. The NSR_HALF database comprises 24 recordings randomly chosen from the NSR database. The CHF_HALF database comprises 14 recordings chosen from the CHF database. Two tests were conducted: one comparing the two original databases and another involving post-hoc analysis comparing all four databases. In both cases, three significance level correction strategies were compared: Bonferroni, FDR, and no correction. The four databases and an R Markdown document for reproducing the results of this section are available at [37].

When comparing only the NSR and CHF databases with any of the three methods, the indices SDNN, SDANN, SDNNIDX, IRRR, TINN, HRVi, ULF, VLF present statistically significant differences. The LF index also presents differences if the correction is made with FDR (p-value of 0.025) or if no correction is applied (p-value of 0.016), but not when Bonferroni is used (p-value of 0.2312). If the non-linear indices are also computed, the p-values for the time and frequency-based indices change slightly due to the additional comparisons, but the same time and frequency indices remain significant. In addition, Poincaré’s *SD*_2_ and six RQA indices (recurrence, divergence, maximal line lengths of vertical and horizontal lines, laminarity, and trapping time) present statistically significant differences. The RQA trend and the averaged diagonal line length are also significant when ignoring corrections (p-values of 0.0199 and 0.0060) or when using FDR (p-values of 0.0325 and 0.0115). The RQA entropy and the correlation dimension are also significant if no correction is applied (p-values of 0.0491 and 0.0391).

It was expected that significant differences would be found in many indices since a database of healthy subjects is being compared with a database of patients with a severe heart condition. It is known that patients with CHF present marked differences in the power of the VLF spectral band, and lesser differences in LF, but no differences in the HF band [38]. The findings about the temporal indices are also consistent with the scientific literature [39, 40]. Regarding the non-linear analysis, although there is less consensus in the literature, there are some authors who show that the indices derived from RQA tend to present differences between healthy subjects and patients with chronic congestive failure [41]. Furthermore, *SD*_2_ is related to long-term variations in the RR intervals, thus the fact that this index presents differences (and not *SD*_1_, which is related to short-term variations) is consistent with the differences found in the low and very low frequency bands (VLF and LF).

When comparing the four databases (NSR, NSR_HALF, CHF, and CHF_HALF) post-hoc tests yielded the expected results, as summarised in Table 1. Significant differences were observed between the pairs NSR and CHF, NSR and CHF_HALF, NSR_HALF and CHF, and NSR_HALF and CHF_HALF, regardless the correction method employed. No significant differences were observed between the pairs NSR and NSR_HALF, and CHF and CHF_HALF for none of the indices and any of the correction methods.

**Table 1:** Number of significant indices in post-hoc analysis using different adjustment methods.

Adj. method	None	FDR	Bonferroni
Comparison			
NSR vs. CHF	18	16	13
NSR vs. CHF_HALF	15	15	12
NSR vs. NSR_HALF	0	0	0
NSR_HALF vs. CHF	18	16	12
NSR_HALF vs. CHF_HALF	15	15	8
CHF vs. CHF_HALF	0	0	0

The number of significant indices varied depending on the correction method, with no-correction yielding the highest number: up to 18 indices in the comparison of the complete databases, and between 15 and 18 when the comparisons involve the sampled databases (see Table 1). When the method used was FDR, 16 indices presented significant differences between the complete databases, and between 15 and 16 in the other cases; this represents a slight decrease compared to the application of no correction method. With Bonferroni the number of differences decreased noticeably: 13 indices for the complete databases, and between 8 and 12 in the other cases.

In all cases, the number of significant indices decreases with the sample size of the compared groups. This was expected since decreasing the sample size lowers the statistical power. The NSR vs. CHF comparison had the maximum number of significant indices (e.g., 13 with Bonferroni), while the minimum was observed in NSR_HALF vs. CHF_HALF (8 indices). Notably, significant indices in all instances were a subset of those identified in the NSR vs. CHF comparison.

Using the NSR and CHF databases, the mean computation time per file (without any parallelization) was 6.02 ± 4.77 minutes on a computer equipped with an AMD Ryzen Threadripper 2970WX, operating at a maximum frequency of 3GHz. The longest computation time observed was 24.12 minutes. The non-linear analysis indices, on average, account for 99.8% of the total computation time. When only the time and frequency indices were computed, the computation time per file decreased to 0.43 ± 0.10 seconds per file.

## 4 Discussion and conclusions

The R open-source package *RHRVEasy* performs a complete HRV analysis by simply calling a function with a single parameter: a list of two or more folders containing the recordings of the different populations of the study. Although it is not necessary to specify any additional parameters to perform the analysis, the default parameter values used in the *RHRV* package can be overridden in the function call. The *RHRVEasy* package preprocesses the RR recordings and computes up to 31 time, frequency, and non-linear HRV indices. Notably, it automates the computation of non-linear indices, which typically requires manual intervention. Then, it performs the statistical analysis, which includes hypothesis testing, significance level correction and, if there are more than two experimental groups, post-hoc analysis. The results of all the statistical tests, as well as the value of every index for each recording of each group, are available in the object returned by the RHRVEasy function. Hence, *RHRVEasy* greatly simplifies performing HRV analysis.

The tests performed, available in the package’s GitHub repository as an R Markdown document [37], have produced the expected results. On the one hand, the statistically significant differences found for the temporal, frequency, and non-linear indices are consistent with previous results in the literature [38–41]. On the other hand, in the tests involving databases NSR, CHF, NSR_HALF and CHF_HALF, no statistically significant differences were ever found between an original database and the database generated by sampling the original one. The number of statistically significant differences decreased with the sample size (see Table 1) and when using more aggressive significance correction methods, decreasing from 16 indices (FDR) to 13 (Bonferroni) for comparisons between the complete databases, and from 15 to 8 for the sampled databases.

In 4 recordings of the CHF database and 2 of the NSR database it was not possible to calculate SDANN and SDNNIDX. For both databases, it was possible to calculate all the non-linear statistics for all the recordings. This required considerable effort in the *RHRVEasy* package; in the early implementations, the non-linear calculations failed for about a third of the non-linear statistics. During the development of the package, the techniques for dealing with the different errors that could arise were gradually improved and refined. For example, up to 5 different strategies are attempted to estimate the time lag that will be used to reconstruct the phase space. However, despite the success in the calculations over these two databases, it should be noted that it is possible that all these error correction strategies fail for some recordings and that some non-linear statistics cannot be calculated.

*RHRVEasy* is a valuable tool to compute a comprehensive set of common HRV indices and to automatically conduct statistical analyses. This makes it a great tool for exploratory studies, where researchers seek insights into a new situation or when no specific hypothesis exists. In this scenario, *RHRVEasy* may help identify patterns or generate hypotheses for subsequent confirmatory studies. However, it can also be utilized in a confirmatory study. Alongside its automated computation of indices, it offers users the flexibility to selectively choose a subset of indices for comparison, empowering researchers to concentrate on specific indices for which they hypothesize differences.

Although the package *RHRVEasy* considerably simplifies HRV analysis, some considerations must also be taken into account in its usage. The parameters used by default to calculate the different indices are those typically employed in studies carried out on adult humans at rest or when performing non-intense physical exercise. Adjustments, especially in parameters related to spectral analysis, are necessary when analyzing data from children [42], athletes [43], or animals [44, 45]. Furthermore, the optimal calculation of some non-linear indices requires the analyst to visually inspect plots to make decisions about the parameters to be used for each recording. Therefore, the values of these non-linear indices provided by the package *RHRVEasy* should be considered approximations. They may be well suited for exploratory analysis, but if any finding related to these indices is found, it should be confirmed by selecting the appropriate parameters based on the visual inspection of the plots for each recording.

The open-source *RHRVEasy* package is freely available under the GPL-2 license. Its GitHub repository (https://github.com/constantino-garcia/RHRVEasy) facilitates community collaboration, enabling users to report bugs and suggest extensions that meet their evolving needs. In future developments, we will integrate new indices into *RHRVEasy*, such as the 1/f slope and detrended fluctuation analysis, which are currently available in *RHRV*. Additionally, we aim to empower users to incorporate custom HRV indices into the statistical analysis provided by *RHRVEasy*. Lastly, we plan to enhance *RHVVEasy* with a Shiny-based user interface, which may contribute to its wider adoption.

## Supporting information

S1 Table Time-domain and frequency-domain HRV indices included in *RHRVEasy*. (ZIP)

S2 Table Non-linear HRV indices included in *RHRVEasy*. (ZIP)

S1 Appendix Experimental databases. (ZIP)

S1 File (ZIP)
