# Supplementary material for: RHRVEasy: Heart rate variability made easy
Source: PLoS One. 2024 Nov 27;19(11):e0309055. doi: 10.1371/journal.pone.0309055 (PMC11602035; doi:10.1371/journal.pone.0309055)
Supplement: S1 Table — (ZIP) [file pone.0309055.s001.zip › S1 Table.pdf]

S1 Table

Table 1. Time-domain and frequency-domain HRV indices included in *RHRVEasy*. See [1,2] for an overview of these methods. Specific references used in the implementation are also cited as needed in the *description*.

**Index (units, if applicable)**	**Type**	**Description**	**Default parameter values in *RHRVEasy***
SDNN (ms)	Time	Standard deviation of all NN intervals	
SDANN (ms)	Time	Standard deviation of the averages of NN intervals in all segments of length `window` (in seconds) of the entire recording	`window = 300`
RMSSD (ms)	Time	The square root of the mean of the sum of the squares of differences between adjacent NN intervals	
SDNN index (ms)	Time	Mean of the standard deviations of all NN intervals for all segments of length `window` (in seconds) of the entire recording	`window = 300`
MADRR (ms)	Time	Median of the absolute differences between adjacent NN intervals	
SDSD (ms)	Time	Standard deviation of differences between adjacent NN intervals	
IRRR (ms)	Time	Interquantile difference of the NN intervals in the entire recording	
pNN50	Time	Percentage of number of pairs of adjacent NN intervals differing by more than 50 ms in the entire recording	
HRVi	Time	HRV triangular index: the total number of all NN intervals divided by the height of the histogram of the NN intervals, measured on a discrete scale with bins of size `interval` ms	`interval=7.8125`
TINN (ms)	Time	Baseline width of the histogram of all NN intervals, determined by triangular interpolation of the highest peak. This is measured using bins of size `interval` ms (`interval` = 7.8125)	`interval=7.8125`
ULF power (ms^2)	Frequency	Absolute power of the Ultra-Low-Frequency band (`ULFmin`, `ULFmax`). Fourier-based methods are used by default. If wavelets are used, the mother wavelet (`wavelet`) and the band-tolerance (`bandtolerance`) [3] should also be specified	`ULFmin = 0,` `ULFmax = 0.03,` `typeAnalysis="fourier",` `wavelet="d4",` `bandtolerance=0.01`
VLF power (ms^2)	Frequency	Absolute power of the Very-Low-Frequency band (`VLFmin`, `VLFmax`)	`VLFmin = 0.03,` `VLFmax = 0.05,` `typeAnalysis="fourier",` `wavelet="d4",` `bandtolerance=0.01`
LF power (ms^2)	Frequency	Absolute power of the Low-Frequency band (`LFmin`, `LFmax`)	`LFmin = 0.05,` `LFmax = 0.15,` `typeAnalysis="fourier",` `wavelet="d4",` `bandtolerance=0.01`
HF power (ms^2)	Frequency	Absolute power of the Figh-Frequency band (`HFmin`, `HFmax`)	`HFmin = 0.15,` `HFmax = 0.4,` `typeAnalysis="fourier",` `wavelet="d4",` `bandtolerance=0.01`

LF/HF	Frequency	Ratio of LF-to-HF power	`LFmin = 0.05, LFmax = 0.15, HFmin = 0.15, HFmax = 0.4, typeAnalysis="fourier", wavelet="d4", bandtolerance=0.01`

References

[1] Electrophysiology, Task Force of the European Society of Cardiology the North American Society of Pacing. Heart rate variability: standards of measurement, physiological interpretation, and clinical use. Circulation. 1996;93(5):1043–1065.

[2] Shaffer F, Ginsberg J. An overview of heart rate variability metrics and norms. Frontiers in public health. 2017;5:258.

[3] García CA, Otero A, Vila X, Márquez DG. A new algorithm for wavelet-based heart rate variability analysis. Biomedical Signal Processing and Control. 2013;8(6):542–550.
