# Supplementary material for: RHRVEasy: Heart rate variability made easy
Source: PLoS One. 2024 Nov 27;19(11):e0309055. doi: 10.1371/journal.pone.0309055 (PMC11602035; doi:10.1371/journal.pone.0309055)
Supplement: S2 Table — (ZIP) [file pone.0309055.s002.zip › S2 Table.pdf]

S2 Table

Table 1. Non-linear HRV indices included in *RHRVEasy*. Although Recurrence Quantification Analysis (RQA) is a non-linear technique, we differentiate it from other non-linear techniques in the *type* column. Most of these indices rely on the calculation of a suitable embedding dimension (noted as E); a time-lag (L); and an estimate of a small radius (R) (see main text for details on the calculation of their values). See [1] for a general overview of non-linear methods and [2] for RQA, [3] for their application to HRV, and [4,5] for the *RHRV* implementation of these methods. Specific references used in the implementation are also cited as needed in the *description*.

**Index (units, if applicable)**	**Type**	**Description**	**Default values in *RHRVEasy***
Poincaré plot's SD1 (ms)	Non-linear	Standard deviation perpendicular to the line of identity in the Poincaré plot, indicating short-term HRV [6]	
Poincaré plot's SD2 (ms)	Non-linear	Standard deviation along the line of identity in the Poincaré plot, representing long-term HRV [6]	
Correlation dimension	Non-linear	The correlation dimension estimates the minimum number of variables required to describe the underlying dynamics. The range of radii considered for computations ranges from `minRadius` to `maxRadius` (in ms). A Theiler window of `theiler` steps is used to exclude temporally correlated points	L, E, `minRadius=1`, `maxRadius=100`, `theiler=10`.
Sample Entropy	Non-linear	The Sample entropy measures the regularity and complexity of a time series	L, E, `minRadius=1`, `maxRadius=100`, `theiler=10`
Maximum Lyapunov exponent	Non-linear	The maximum Lyapunov exponent measures the rate at which small differences in HRV grow, indicating the system's sensitivity to initial conditions and overall chaotic behavior [7]. The divergence rate of close trajectories is computed for `numberTimeSteps` time steps	L, E, R, `theiler=10`, `numberTimeSteps=20`
Recurrence	RQA	Percentage of recurrent points in a recurrence plot, representing the system's tendency to return to previous states. To avoid border effects, the `distanceToBorder` points near the border of the recurrence matrix are ignored when computing the RQA parameters	L, E, R, `distanceToBorder=2`
Determinism	RQA	Percentage of recurrent points forming diagonal lines in a recurrence plot, indicating the presence of deterministic patterns. A minimal length of `lmin` is required to a line to be considered in the RQA calculations	L, E, R, `lmin=2`, `distanceToBorder=2`,
Laminarity	RQA	Percentage of recurrent points forming vertical lines in a recurrence plot, reflecting periods where the system remains in a particular state. A minimal length of `vmin` is required to a line to be considered in the RQA calculations	L, E, R, `vmin=2`
Ratio	RQA	Ratio of *determinism* to *recurrence*. That is, the ratio between the number of recurrent points in diagonal lines and the total number of points of the recurrence matrix	L, E, R, `lmin=2`, `distanceToBorder=2`
Averaged diagonal line length	RQA	Mean length of diagonal lines in a recurrence plot, related to the average time two close states remain correlated	L, E, R, `lmin=2`, `distanceToBorder=2`
Trapping time	RQA	Average length of vertical lines in a recurrence plot, indicating the mean time the system remains in a specific state	L, E, R, `vmin=2`, `distanceToBorder=2`
Longest diagonal line	RQA	Length of the longest diagonal line in a recurrence plot, inversely related to the maximum Lyapunov exponent	L, E, R, `lmin=2`, `distanceToBorder=2`

Divergence	RQA	Inverse of the longest diagonal line length, scaling with the maximum Lyapunov exponent to measure system divergence	L, E, R, `lmin=2`, `distanceToBorder=2`
Longest vertical line	RQA	Maximum length of vertical lines in a recurrence plot, representing the longest time the system remains in one state	L, E, R, `vmin=2`, `distanceToBorder=2`
Entropy	RQA	Shannon entropy of the diagonal line lengths distribution	L, E, R,`lmin=2`, `distanceToBorder=2`
Trend	RQA	Trend in the number of recurrent points based on their distance from the main diagonal in a recurrence plot	L, E, R, `distanceToBorder=2`

References

[1] Kantz H, Schreiber T. Nonlinear time series analysis. vol. 7. Cambridge university press; 2004.

[2] Zbilut JP, Webber Jr CL. Recurrence quantification analysis. Wiley encyclopedia of biomedical engineering. 2006;.

[3] Voss A, Schulz S, Schroeder R, Baumert M, Caminal P. Methods derived from nonlinear dynamics for analysing heart rate variability. Philosophical Transactions of the Royal Society A: Mathematical, Physical and Engineering Sciences. 2009;367(1887):277–296.

[4] García CA, Otero A, Presedo J, Vila X, Félix P. A software toolkit for nonlinear Heart Rate Variability analysis. In: Computing in Cardiology 2013. IEEE; 2013. p. 393–396.

[5] García CA, Quintana AO, Vila XA, Touriño MJL, Rodríguez-Liñares L, Presedo JMR, et al. Heart rate variability analysis with the R package RHRV. Springer; 2017.

[6] Shaffer F, Ginsberg J. An overview of heart rate variability metrics and norms. Frontiers in public health. 2017;5:258.

[7] Rosenstein MT, Collins JJ, De Luca CJ. A practical method for calculating largest Lyapunov exponents from small data sets. Physica D: Nonlinear Phenomena. 1993;65(1-2):117–134.
