# Supplementary material for: RHRVEasy: Heart rate variability made easy
Source: PLoS One. 2024 Nov 27;19(11):e0309055. doi: 10.1371/journal.pone.0309055 (PMC11602035; doi:10.1371/journal.pone.0309055)
Supplement: S1 Appendix — (ZIP) [file pone.0309055.s003.zip › S3 Appendix.pdf]

S3 Appendix

Experimental databases

Healthy subjects' recordings were obtained from the Normal Sinus Rhythm (NSR) RR Interval Database [1]. This database comprises 54 long-term recordings (approximately 24 hours) of 30 men aged 28 to 76 and 24 women aged 58 to 73. Pathological recordings were obtained from the Congestive Heart Failure (CHF) RR Interval Database [1], consisting of 29 long-term recordings (also around 24 hours) of subjects aged 34 to 79, including 8 men and 2 women; the gender of the remaining subjects is unknown. The original ECG recordings from both databases were digitized at 128 samples per second, with beat annotations obtained through automated analysis followed by manual review and correction.

References

[1] Goldberger AL, Amaral LA, Glass L, Hausdorff JM, Ivanov PC, Mark RG, et al. PhysioBank, PhysioToolkit, and PhysioNet: components of a new research resource for complex physiologic signals. circulation. 2000;101(23):e215–e220.
