# Supplementary material for: RHRVEasy: Heart rate variability made easy
Source: PLoS One. 2024 Nov 27;19(11):e0309055. doi: 10.1371/journal.pone.0309055 (PMC11602035; doi:10.1371/journal.pone.0309055)
Supplement: S1 File — (ZIP) [file pone.0309055.s004.zip › RHRV-submission/documentation/RHRVEasyTutorial.html]

RHRVEasy tutorial


# RHRVEasy tutorial

- 0. Set up required to run
  this tutorial
- 1. Time and frequency
  analysis
- 2. Correction of the
  significance level
- 3. Saving the
  indices to an Excel spreadsheet
- 4. Comparing more
  than two experimental groups
- 5. Overwriting default
  parameters
- 6.
  Nonlinear analysis

RHRVEasy automates all steps of a Heart Rate Variability (HRV)
analysis, including data processing, indices calculation, and
statistical analysis. It takes as input a list of folders, each
containing the recordings of a same population. It calculates time,
frequency, and nonlinear domain HRV indices, and then it applies
hypothesis test, and corrects the significance levels. If there are more
than two experimental groups and statistically significant differences
are found, it performs a post-hoc analysis to find out which groups have
the differences.

# 0. Set up required to run this tutorial

This tutorial uses the recordings of the Normal Sinus Rhythm
RR Interval Database (hereinafter referred to as NSR\_DB) and of the
Congestive
Heart Failure RR Interval Database (hereinafter referred to as
CHF\_DB). The first is made of healthy subjects, and the second of
patients with a severe cardiac pathology. Hence significant differences
in many HRV indices are expected between them.

To illustrate how to work with more than two experimental groups,
half of the recordings from both databases were randomly selected. The
NSR\_HALF\_DB database is made up of 24 recordings randomly chosen from
the 58 of the NSR database. The CHF\_HALF\_DB database is made up of 14
recordings chosen from the 28 of the CHF database. The four databases
are available in the RHRVEasy GitHub
repository. To run this tutorial, the following four variables must
contain the folder of the local machine where the files of each of these
databases are

```
library("RHRVEasy")

basePath = "RRData/" 
NSR_DB = file.path(basePath, "normal")
CHF_DB = file.path(basePath, "chf")
NSR_HALF_DB = file.path(basePath, "normal_half")
CHF_HALF_DB = file.path(basePath, "chf_half")
```

RHRVEasy permits creating an Excel spreadsheet with all the HRV
indices calculated for each recording. The following variable must
contain the folder on the local machine where the Excel spreadsheet is
to be saved:

```
spreadsheetPath = basePath
```

# 1. Time and frequency analysis

`RHRVEasy` enables the user to carry out a full HRV
analysis by just invoking a function with a single mandatory parameter:
a list with the folders containing the recordings of the experimental
groups. This list must have at least two folders. Each folder must
contain all the RR recordings of the same experimental group and no
additional files, as `RHRVEasy` will try to open all the
files in those folders. The name that will be used to refer to each
experimental group within `RHRVEasy` will be the name of the
folder in which its recordings are located.

The following function call computes the time and frequency indices
for the NSR\_DB and CHF\_DB databases, and and performs a statistical
comparison of each index correcting the significance level with the
Bonferroni method. Note the use of the `nJobs` to use several
cores and parallelize the computations.

```
easyAnalysis = RHRVEasy(folders = c(NSR_DB, CHF_DB), nJobs = -1)
```

When the returned object is displayed in the console, it shows which
indices present statistically significant differences:

```
print(easyAnalysis)
```

```
## Significant differences in SDNN (Kruskal-Wallis rank sum test, bonferroni p-value = 1.117154e-07):
##   chf's mean95% CI: (63.0561, 92.59621) [Bootstrap CI without adjustment]
##   normal's mean95% CI: (131.851, 148.7659) [Bootstrap CI without adjustment]
## 
## Significant differences in SDANN (Kruskal-Wallis rank sum test, bonferroni p-value = 3.799696e-07):
##   chf's mean95% CI: (47.92118, 80.41882) [Bootstrap CI without adjustment]
##   normal's mean95% CI: (120.6496, 138.2731) [Bootstrap CI without adjustment]
## 
## Significant differences in SDNNIDX (Kruskal-Wallis rank sum test, bonferroni p-value = 0.01426098):
##   chf's mean95% CI: (29.62375, 47.79535) [Bootstrap CI without adjustment]
##   normal's mean95% CI: (46.91484, 54.40014) [Bootstrap CI without adjustment]
## 
## Significant differences in IRRR (Kruskal-Wallis rank sum test, bonferroni p-value = 1.492754e-07):
##   chf's mean95% CI: (78.13184, 123.1074) [Bootstrap CI without adjustment]
##   normal's mean95% CI: (186.2053, 216.4315) [Bootstrap CI without adjustment]
## 
## Significant differences in TINN (Kruskal-Wallis rank sum test, bonferroni p-value = 1.452872e-06):
##   chf's mean95% CI: (246.4746, 375.0049) [Bootstrap CI without adjustment]
##   normal's mean95% CI: (513.6919, 586.3119) [Bootstrap CI without adjustment]
## 
## Significant differences in HRVi (Kruskal-Wallis rank sum test, bonferroni p-value = 1.452872e-06):
##   chf's mean95% CI: (15.95139, 23.98438) [Bootstrap CI without adjustment]
##   normal's mean95% CI: (32.85439, 37.53765) [Bootstrap CI without adjustment]
## 
## Significant differences in ULF (Kruskal-Wallis rank sum test, bonferroni p-value = 1.74099e-08):
##   chf's mean95% CI: (1140.113, 4373.228) [Bootstrap CI without adjustment]
##   normal's mean95% CI: (6962.452, 9865.318) [Bootstrap CI without adjustment]
## 
## Significant differences in VLF (Kruskal-Wallis rank sum test, bonferroni p-value = 0.002535127):
##   chf's mean95% CI: (47.22639, 136.2537) [Bootstrap CI without adjustment]
##   normal's mean95% CI: (130.731, 174.1791) [Bootstrap CI without adjustment]
```

All computed indices, as well as all p-values resulting from all
comparisons, are stored in `data.frames` contained in the
object. Two different sets of p-values are available; the ones obtained
before (`p.value`) and after (`adj.p.value`)
applying the significance level correction:

```
# HRVIndices
head(easyAnalysis$HRVIndices)
```

```
##                 file group     SDNN    SDANN  SDNNIDX    pNN50     SDSD
## 1 chf201_rr_secs.txt   chf 75.50523 52.90834 49.58724 2.026744 20.21072
## 2 chf202_rr_secs.txt   chf 88.53389 75.77262 39.59771 6.134879 34.71964
## 3 chf203_rr_secs.txt   chf 38.77109 30.87307 21.72908 1.198474 17.27104
## 4 chf204_rr_secs.txt   chf 55.13204 39.05113 36.00899 4.838509 33.02579
## 5 chf205_rr_secs.txt   chf 34.91304 26.09401 19.54139 1.966332 23.69980
## 6 chf206_rr_secs.txt   chf 41.22358 34.90781 14.84015 2.022305 18.93981
##      rMSSD     IRRR   MADRR     TINN      HRVi       ULF        VLF        LF
## 1 20.21063  93.7500  7.8125 357.6871 22.891975 2528.0667 122.009599 161.67723
## 2 34.71948 117.1875 15.6250 350.4726 22.430249 1855.7710 104.542314 281.24798
## 3 17.27095  46.8750  7.8125 170.2116 10.893544  620.7563   6.047730  11.65002
## 4 33.02562  70.3125  7.8125 237.0307 15.169962 1085.5905  29.484504  77.44332
## 5 23.69971  46.8750  7.8125 168.6735 10.795107  528.7881  43.922773  84.91362
## 6 18.93974  31.2500  7.8125 121.7600  7.792642  587.5320   3.697945  11.21686
##          HF
## 1  58.79672
## 2 140.84916
## 3  19.15383
## 4  80.86120
## 5  51.94413
## 6  17.06285
```

```
# Statistical analysis
head(easyAnalysis$stats)
```

```
## # A tibble: 6 × 4
##         p.value method                       HRVIndex adj.p.value
##           <dbl> <chr>                        <chr>          <dbl>
## 1 0.00000000798 Kruskal-Wallis rank sum test SDNN     0.000000112
## 2 0.0000000271  Kruskal-Wallis rank sum test SDANN    0.000000380
## 3 0.00102       Kruskal-Wallis rank sum test SDNNIDX  0.0143     
## 4 0.774         Kruskal-Wallis rank sum test pNN50    1          
## 5 0.0891        Kruskal-Wallis rank sum test SDSD     1          
## 6 0.0891        Kruskal-Wallis rank sum test rMSSD    1
```

The `format` parameter specifies the format in which the
RR intervals are stored. All formats supported by the RHRV package can
be used: `WFDB`, `ASCII`, `RR`,
`Polar`, `Suunto`, `EDFPlus` or
`Ambit` (check the RHRV website for more
information). The default format is RR, where the beat distances in
seconds are stored in a single column of an ASCII file. This is the
format of the four databases used in this tutorial.

By default, the frequency analysis is performed using the Fourier
transform. It is also possible to use the Wavelet transform pasing the
value `'wavelet'` to the `typeAnalysis` parameter
(check the paper “García, C. A., Otero, A., Vila, X., & Márquez, D.
G. (2013). A new algorithm for wavelet-based heart rate variability
analysis. Biomedical Signal Processing and Control, 8(6), 542-550” for
details):

```
easyAnalysisWavelet = RHRVEasy(
  folders = c(NSR_DB, CHF_DB), 
  typeAnalysis = 'wavelet', 
  n_jobs = -1
)
```

The results are similar to the previous ones:

```
print(easyAnalysisWavelet)
```

```
## Significant differences in SDNN (Kruskal-Wallis rank sum test, bonferroni p-value = 1.117154e-07):
##   chf's mean95% CI: (63.50081, 93.35745) [Bootstrap CI without adjustment]
##   normal's mean95% CI: (131.3645, 147.8141) [Bootstrap CI without adjustment]
## 
## Significant differences in SDANN (Kruskal-Wallis rank sum test, bonferroni p-value = 3.799696e-07):
##   chf's mean95% CI: (48.26282, 80.92454) [Bootstrap CI without adjustment]
##   normal's mean95% CI: (120.9452, 138.4027) [Bootstrap CI without adjustment]
## 
## Significant differences in SDNNIDX (Kruskal-Wallis rank sum test, bonferroni p-value = 0.01426098):
##   chf's mean95% CI: (30.15555, 47.39782) [Bootstrap CI without adjustment]
##   normal's mean95% CI: (46.63857, 54.4691) [Bootstrap CI without adjustment]
## 
## Significant differences in IRRR (Kruskal-Wallis rank sum test, bonferroni p-value = 1.492754e-07):
##   chf's mean95% CI: (78.125, 124.185) [Bootstrap CI without adjustment]
##   normal's mean95% CI: (187.793, 216.2832) [Bootstrap CI without adjustment]
## 
## Significant differences in TINN (Kruskal-Wallis rank sum test, bonferroni p-value = 1.452872e-06):
##   chf's mean95% CI: (250.0613, 375.947) [Bootstrap CI without adjustment]
##   normal's mean95% CI: (508.9647, 584.8309) [Bootstrap CI without adjustment]
## 
## Significant differences in HRVi (Kruskal-Wallis rank sum test, bonferroni p-value = 1.452872e-06):
##   chf's mean95% CI: (15.82573, 24.10886) [Bootstrap CI without adjustment]
##   normal's mean95% CI: (32.47397, 37.43171) [Bootstrap CI without adjustment]
## 
## Significant differences in ULF (Kruskal-Wallis rank sum test, bonferroni p-value = 2.341794e-08):
##   chf's mean95% CI: (1053029673, 3162782925) [Bootstrap CI without adjustment]
##   normal's mean95% CI: (5700337997, 7578178863) [Bootstrap CI without adjustment]
## 
## Significant differences in VLF (Kruskal-Wallis rank sum test, bonferroni p-value = 0.003692366):
##   chf's mean95% CI: (27615755, 75218273) [Bootstrap CI without adjustment]
##   normal's mean95% CI: (71189420, 93315386) [Bootstrap CI without adjustment]
```

# 2. Correction of the significance level

Given that multiple statistical tests are performed on several HRV
indices, a correction of the significance level should be applied. The
Bonferroni method is used by default. This behavior can be overridden
with the parameter `correctionMethod` of
`RHRVEasy`. The possible values of this parameter besides
`bonferroni` are `holm`, `hochberg`,
`hommel`, `BH` (Benjamini & Hochberg),
`fdr` (false discovery rate), `BY` (Benjamini
& Yekutieli), and `none` (indicating that no correction
is to be made). Furthermore, there is no need to recompute the HRV
indices to apply a different correction method, but the
`RHRVEasyStats` function. The confidence level can also be
changed using the `significance` parameter (in both
`RHRVEasy` and `RHRVEasyStats` functions).

```
easyAnalysisFDR = RHRVEasyStats(easyAnalysis, correctionMethod =  'fdr')
pValues = merge(
  easyAnalysis$stats, 
  easyAnalysisFDR$stats,
  by = setdiff(names(easyAnalysis$stats), "adj.p.value"),
  suffixes = c(".bonf", ".fdr")
)
#Let us compare the p-values obtained with different correction methods 
print(
  head(
    pValues[, c("HRVIndex", "p.value", "adj.p.value.bonf", "adj.p.value.fdr")]
  )
)
```

```
##   HRVIndex      p.value adj.p.value.bonf adj.p.value.fdr
## 1      VLF 0.0001810805      0.002535127    0.0003621611
## 2  SDNNIDX 0.0010186412      0.014260976    0.0017826220
## 3       LF 0.0165147930      0.231207102    0.0256896781
## 4    MADRR 0.0631990263      0.884786369    0.0884786369
## 5    rMSSD 0.0891116490      1.000000000    0.1039635905
## 6     SDSD 0.0891116490      1.000000000    0.1039635905
```

# 3. Saving the indices to an Excel spreadsheet

If the argument `saveHRVindicesInPath` is specified when
invoking the function `RHRVEasy`, an Excel spreadsheet with
all the HRV indices calculated for each recording will be created in the
path specified specified in this parameter. The name of the spreadsheet
generated is “<group 1 name>*Vs*<group 2 name>
.xlsx”:

```
easyAnalysis = RHRVEasy(folders = c(NSR_DB, CHF_DB), 
                        saveHRVIndicesInPath = spreadsheetPath)
```

This spreadsheet can also be generated from the object returned by
`RHRVEasy` by calling the function
`saveHRVIndices`.

```
saveHRVIndices(easyAnalysis,saveHRVIndicesInPath = spreadsheetPath)
```

# 4. Comparing more than two experimental groups

If the analysis involves three or more groups, when statistically
significant differences are found among them it does not necessarily
mean that there are statistically significant differences between all
pairs of groups. In such a scenario the Dunn post-hoc is used to find
which pairs of groups present differences:

```
#Comparison of the four databases
easyAnalysis4 = RHRVEasy(
  folders = c(NSR_DB, CHF_DB, NSR_HALF_DB, CHF_HALF_DB),
  nJobs = -1
)
```

```
print(easyAnalysis4)
```

```
## Significant differences in SDNN (Kruskal-Wallis rank sum test, bonferroni p-value = 4.456566e-09):
##   Significant differences in the post-hoc tests (Dunn's all-pairs test + bonferroni-p-value adjustment):
##       group1      group2   adj.p.value
##     1 normal      chf      0.000000979
##     2 normal      chf_half 0.000725   
##     3 normal_half chf      0.0000729  
##     4 normal_half chf_half 0.00352    
##     ----------------------------------
##     chf's mean95% CI: (62.72614, 94.55918) [Bootstrap CI without adjustment]
##     chf_half's mean95% CI: (52.37089, 96.6655) [Bootstrap CI without adjustment]
##     normal's mean95% CI: (131.4973, 147.8025) [Bootstrap CI without adjustment]
##     normal_half's mean95% CI: (127.8219, 157.0621) [Bootstrap CI without adjustment]
## 
## Significant differences in SDANN (Kruskal-Wallis rank sum test, bonferroni p-value = 1.942718e-08):
##   Significant differences in the post-hoc tests (Dunn's all-pairs test + bonferroni-p-value adjustment):
##       group1      group2   adj.p.value
##     1 normal      chf       0.00000297
##     2 normal      chf_half  0.00209   
##     3 normal_half chf       0.0000845 
##     4 normal_half chf_half  0.00557   
##     ----------------------------------
##     chf's mean95% CI: (48.52008, 81.35924) [Bootstrap CI without adjustment]
##     chf_half's mean95% CI: (38.30645, 83.27054) [Bootstrap CI without adjustment]
##     normal's mean95% CI: (121.0169, 138.5931) [Bootstrap CI without adjustment]
##     normal_half's mean95% CI: (118.2212, 149.916) [Bootstrap CI without adjustment]
## 
## Significant differences in SDNNIDX (Kruskal-Wallis rank sum test, bonferroni p-value = 0.009806272):
##     No significant differences were found between groups in post-hoc tests ( Dunn's all-pairs test  +  bonferroni -p-value adjustment).
## 
## Significant differences in IRRR (Kruskal-Wallis rank sum test, bonferroni p-value = 1.375278e-08):
##   Significant differences in the post-hoc tests (Dunn's all-pairs test + bonferroni-p-value adjustment):
##       group1      group2   adj.p.value
##     1 normal      chf       0.00000111
##     2 normal      chf_half  0.00135   
##     3 normal_half chf       0.000191  
##     4 normal_half chf_half  0.0105    
##     ----------------------------------
##     chf's mean95% CI: (79.47198, 122.2992) [Bootstrap CI without adjustment]
##     chf_half's mean95% CI: (56.36161, 138.9367) [Bootstrap CI without adjustment]
##     normal's mean95% CI: (189.8185, 215.9975) [Bootstrap CI without adjustment]
##     normal_half's mean95% CI: (179.3702, 228.4991) [Bootstrap CI without adjustment]
## 
## Significant differences in TINN (Kruskal-Wallis rank sum test, bonferroni p-value = 1.743324e-07):
##   Significant differences in the post-hoc tests (Dunn's all-pairs test + bonferroni-p-value adjustment):
##       group1      group2   adj.p.value
##     1 normal      chf       0.00000727
##     2 normal      chf_half  0.00766   
##     3 normal_half chf       0.000305  
##     4 normal_half chf_half  0.0244    
##     ----------------------------------
##     chf's mean95% CI: (244.0797, 374.0042) [Bootstrap CI without adjustment]
##     chf_half's mean95% CI: (203.0794, 421.3341) [Bootstrap CI without adjustment]
##     normal's mean95% CI: (514.2064, 588.3338) [Bootstrap CI without adjustment]
##     normal_half's mean95% CI: (504.2522, 584.7393) [Bootstrap CI without adjustment]
## 
## Significant differences in HRVi (Kruskal-Wallis rank sum test, bonferroni p-value = 1.743324e-07):
##   Significant differences in the post-hoc tests (Dunn's all-pairs test + bonferroni-p-value adjustment):
##       group1      group2   adj.p.value
##     1 normal      chf       0.00000727
##     2 normal      chf_half  0.00766   
##     3 normal_half chf       0.000305  
##     4 normal_half chf_half  0.0244    
##     ----------------------------------
##     chf's mean95% CI: (15.62564, 23.85298) [Bootstrap CI without adjustment]
##     chf_half's mean95% CI: (13.22704, 27.243) [Bootstrap CI without adjustment]
##     normal's mean95% CI: (32.90309, 37.42586) [Bootstrap CI without adjustment]
##     normal_half's mean95% CI: (32.11445, 37.5134) [Bootstrap CI without adjustment]
## 
## Significant differences in ULF (Kruskal-Wallis rank sum test, bonferroni p-value = 1.8806e-10):
##   Significant differences in the post-hoc tests (Dunn's all-pairs test + bonferroni-p-value adjustment):
##       group1      group2   adj.p.value
##     1 normal      chf      0.000000118
##     2 normal      chf_half 0.000147   
##     3 normal_half chf      0.0000180  
##     4 normal_half chf_half 0.00103    
##     ----------------------------------
##     chf's mean95% CI: (1166.568, 4471.449) [Bootstrap CI without adjustment]
##     chf_half's mean95% CI: (574.836, 3958.003) [Bootstrap CI without adjustment]
##     normal's mean95% CI: (7138.234, 9917.287) [Bootstrap CI without adjustment]
##     normal_half's mean95% CI: (6300.005, 11726.58) [Bootstrap CI without adjustment]
## 
## Significant differences in VLF (Kruskal-Wallis rank sum test, bonferroni p-value = 0.000836364):
##   Significant differences in the post-hoc tests (Dunn's all-pairs test + bonferroni-p-value adjustment):
##       group1      group2 adj.p.value
##     1 normal      chf         0.0112
##     2 normal_half chf         0.0395
##     --------------------------------
##     chf's mean95% CI: (50.51941, 137.7606) [Bootstrap CI without adjustment]
##     chf_half's mean95% CI: (28.03815, 138.8459) [Bootstrap CI without adjustment]
##     normal's mean95% CI: (130.9205, 176.065) [Bootstrap CI without adjustment]
##     normal_half's mean95% CI: (125.6175, 194.4629) [Bootstrap CI without adjustment]
```

Note that the `stats` `data.frame` now contains
a column named `pairwise` storing the results of the post-hoc
analysis for those indices where the omnibus test has been
significant:

```
print(head(easyAnalysis4$stats))
```

```
## # A tibble: 6 × 5
##    p.value method                       HRVIndex   adj.p.value pairwise        
##      <dbl> <chr>                        <chr>            <dbl> <list>          
## 1 3.18e-10 Kruskal-Wallis rank sum test SDNN     0.00000000446 <tibble [6 × 6]>
## 2 1.39e- 9 Kruskal-Wallis rank sum test SDANN    0.0000000194  <tibble [6 × 6]>
## 3 7.00e- 4 Kruskal-Wallis rank sum test SDNNIDX  0.00981       <tibble [6 × 6]>
## 4 9.71e- 1 Kruskal-Wallis rank sum test pNN50    1             <NULL>          
## 5 4.12e- 1 Kruskal-Wallis rank sum test SDSD     1             <NULL>          
## 6 4.12e- 1 Kruskal-Wallis rank sum test rMSSD    1             <NULL>
```

```
# Let's print the post-hoc comparisons for "SDNN"
print(head(easyAnalysis4$stats$pairwise[[1]]))
```

```
## # A tibble: 6 × 6
##   group1      group2        p.value method                HRVIndex adj.p.value
##   <chr>       <chr>           <dbl> <chr>                 <chr>          <dbl>
## 1 chf_half    chf      0.984        Dunn's all-pairs test SDNN     1          
## 2 normal      chf      0.0000000204 Dunn's all-pairs test SDNN     0.000000979
## 3 normal      chf_half 0.0000151    Dunn's all-pairs test SDNN     0.000725   
## 4 normal_half chf      0.00000152   Dunn's all-pairs test SDNN     0.0000729  
## 5 normal_half chf_half 0.0000733    Dunn's all-pairs test SDNN     0.00352    
## 6 normal_half normal   0.884        Dunn's all-pairs test SDNN     1
```

# 5. Overwriting default parameters

Any parameter of any RHRV function can be specified as an additional
parameter of the `RHRVEasy` function; in this case, the
default value used for that parameter will be overwritten by the one
specified for the user. The default values used in the
`RHRVEasy` package are the same as those used in the RHRV
package. For more information about the parameters available you can
consult the RHRV
website. For example, the following analysis modifies the the limits
of the ULF, VLF, LF and HF spectral bands, and uses an interpolation
frequency (`freqhr`) of 2 Hz:

```
easyAnalysisOverwritten = RHRVEasy(folders = c(NSR_DB, CHF_DB),
                                   freqhr = 2, 
                                   ULFmin = 0, ULFmax = 0.02, 
                                   VLFmin = 0.02,  VLFmax = 0.07, 
                                   LFmin = 0.07, LFmax = 0.20, 
                                   HFmin = 0.20, HFmax = 0.5)
```

# 6. Nonlinear analysis

The calculation of the nonlinear indices requires considerable
computational resources, specially the Recurrence Quantification
Analysis (RQA). Whereas in a typical HRV analysis the computation of all
the time and frequency domain indices for a few dozens of recordings
often completes within a few minutes, the computation of the nonlinear
indices could last many hours. That’s why the boolean parameters
`nonLinear` and `doRQA` are set to
`FALSE` by default. If these parameters are not change, only
time and frequency indices will be calculated, as in the previous
sections.

**Warning**: the following sentence, will take several
hours to execute on a medium to high performance PC. You may reproduce
the results of the paper by running this chunk of code. Alternatively,
you may load the RDS file `RRData/paperExperiments.RDS` to
explore the statistical results.

```
fullAnalysis = RHRVEasy(
  folders = c(NSR_DB, CHF_DB, NSR_HALF_DB, CHF_HALF_DB),
  nJobs = -1,
  nonLinear =  TRUE, 
  doRQA = TRUE,
  saveHRVIndicesInPath = spreadsheetPath
)
```
