# Supplementary material for: RHRVEasy: Heart rate variability made easy
Source: PLoS One. 2024 Nov 27;19(11):e0309055. doi: 10.1371/journal.pone.0309055 (PMC11602035; doi:10.1371/journal.pone.0309055)
Supplement: S1 File — (ZIP) [file pone.0309055.s004.zip › RHRV-submission/documentation/RHRVEasy-manual.pdf]

Package ‘RHRVEasy’

April 29, 2024

Title Simplified statistical analysis of Heart Rate Variability data

Version 0.0.1

Description Runs time-based analysis, frequency-based analysis and non-linear analysis of Heart Rate Variability data with the aim of performing a statistical comparison between several groups (e.g., control and treatment). The user just needs to specify the path to the groups' data files.

License GPL (>= 3)

Encoding UTF-8

Imports boot, broom, doSNOW, foreach, iterators, nonlinearTseries (>= 0.2.13), parallel, plotrix, PMCMRplus, progress, RHRV (>= 2.4.6), segmented, stats, tibble, tidyr, writexl

Suggests knitr, rmarkdown

Roxygen list(markdown = TRUE)

RoxygenNote 7.3.1

NeedsCompilation no

Author Abraham Otero [aut, cre],
Constantino A. Garcia [aut],
Sofía Bardají [aut],
Pablo Pérez-Tirador [aut]

Maintainer Abraham Otero <aotero@ceu.es>

R topics documented:

RHRVEasy 2
RHRVEasyStats 3
saveHRVIndices 4

Index 5

RHRVEasy *Run a full HRV analysis including indices computation and statistical analysis*

Description

Run a full HRV analysis including indices computation and statistical analysis

Usage

```
RHRVEasy(
  folders,
  correctionMethod = c("bonferroni", "holm", "hochberg", "hommel", "BH", "BY", "
    "none"),
  verbose = FALSE,
  format = "RR",
  typeAnalysis = c("fourier", "wavelet"),
  significance = 0.05,
  nonLinear = FALSE,
  doRQA = FALSE,
  nJobs = 1,
  saveHRVIndicesInPath = NULL,
  ...
)
```

Arguments

`folders`	A character vector with the paths to the folders containing the HRV files. Each folder should contain the HRV files of a group.
`correctionMethod`	The method to correct for multiple comparisons. Can be one of "bonferroni", "holm", "hochberg", "hommel", "BH", "BY", "fdr" and "none". Default is "bonferroni".
`verbose`	Logical. If TRUE, the function will show a progress bar and print additional information to the console.
`format`	The format of the HRV files. Can be one of "WFDB", "Ascii", "RR", "Polar", "Suunto", "EDFPlus" and "Ambit".
`typeAnalysis`	The type of frequency analysis to perform. Can be one of "fourier" or "wavelet".
`significance`	The significance level to use in the statistical analysis. By default, it is set to 0.05.
`nonLinear`	Logical. If TRUE, the function will compute non-linear indices. It should be noted that this process is computationally expensive.
`doRQA`	Logical. If TRUE, the function will compute Recurrence Quantification Analysis (RQA) indices. This parameter is ignored if `nonLinear` is set to FALSE.It should be noted that this process is computationally expensive.
`nJobs`	The number of parallel jobs to use. `nJobs <= 0` uses all cores available. By default, it is set to 1.

`saveHRVIndicesInPath`
: The path where the HRV indices will be saved as an excel file. If NULL, the indices will not be saved. See `saveHRVIndices()` for more details.

`...`
: Additional arguments for the HRV analysis. For further details, see the `RHRV` package.

Value

An object of class `RHRVEasyResult` containing the HRV indices (slot `$HRVIndices`) and the statistical analysis results (slot `$stats`)

`RHRVEasyStats` *Rerun the statistical analysis from a previous* `RHRVEasy()` *call*

Description

Rerun the statistical analysis from a previous `RHRVEasy()` call

Usage

```
RHRVEasyStats(
  RHRVEasyResultObject,
  correctionMethod = c("bonferroni", "holm", "hochberg", "hommel", "BH", "BY", "
    "none"),
  significance = 0.05
)
```

Arguments

`RHRVEasyResultObject`
: An object of class `RHRVEasyResult` as returned by `RHRVEasy()`

`correctionMethod`
: The method to correct for multiple comparisons. Can be one of "bonferroni", "holm", "hochberg", "hommel", "BH", "BY", "fdr" and "none". Default is "bonferroni".

`significance`
: The significance level to use in the statistical analysis. By default, it is set to 0.05.

Value

An object of class `RHRVEasyResult` containing the HRV indices (slot `$HRVIndices`) and the statistical analysis results (slot `$stats`)

See Also

`RHRVEasy()`

saveHRVIndices — *Save the HRV indices as an excel file*

Description

Save the HRV indices as an excel file

Usage

```
saveHRVIndices(RHRVEasyResultObject, saveHRVIndicesInPath = ".")
```

Arguments

`RHRVEasyResultObject`
: An object of class `RHRVEasyResult` as returned by `RHRVEasy()`

`saveHRVIndicesInPath`
: The path where the HRV indices will be saved as an excel file. The name of the file is automatically created based on the groups being compared.

Index

RHRVEasy, 2
RHRVEasy(), *3*
RHRVEasyStats, 3

saveHRVIndices, 4
saveHRVIndices(), *3*
