# Supplementary material for: RHRVEasy: Heart rate variability made easy
Source: PLoS One. 2024 Nov 27;19(11):e0309055. doi: 10.1371/journal.pone.0309055 (PMC11602035; doi:10.1371/journal.pone.0309055)
Supplement: S1 File — (ZIP) [file pone.0309055.s004.zip › RHRV-submission/README.html]

README


# RHRVEasy

An R package created to automate all steps of a HRV analysis,
including data preprocessing, indices calculation, and statistical
analysis. The methods of this package are described in:

> García, C.A., Bardají, S., Pérez-Tirador, P., Otero, A.
> **RHRVEasy: heart rate variability made easy**. *Under
> review*

## Installation

### Installing R

1. Go to the R project website
   and download the latest version of R for your operating system. In Linux
   systems, it may be easier to use the package manager to install R (In
   that case, step 2 is not necessary).
2. Install R by following the instructions provided in the website.
   Default options are fine for most users.

### Installing RHRVEasy

There are several options to install the package:

#### Option 1: Install from GitHub

Use `devtools` to install the package. In an R console,
execute the following commands:

```
# install.packages("devtools") # only if needed
devtools::install_github("constantino-garcia/RHRVEasy")
```

#### Option 2: Install from source

Using the RHRVEasy\_XXX.tar.gz file, where XXX is the version of the
package. In an R console, execute the following commands:

```
# Install dependencies
install.packages(c("boot", "broom", "doSNOW", "foreach",
  "iterators", "nonlinearTseries", "plotrix",
  "PMCMRplus", "progress", "RHRV", "segmented", 
  "tibble", "tidyr", "writexl"))
# Install package. Remember to replace path/to/RHRVEasy_XXX.tar.gz with the actual path and 
# version of the package
install.packages("path/to/RHRVEasy_XXX.tar.gz", repos = NULL, type = "source")
```

#### Troubleshooting

In case dependencies are not installed automatically, you can install
them manually by running:

```
install.packages(c("boot", "broom", "doSNOW", "foreach",
  "iterators", "nonlinearTseries", "plotrix",
  "PMCMRplus", "progress", "RHRV", "segmented", 
  "tibble", "tidyr", "writexl"))
```

## API overview

The main function of the package is `RHRVEasy` and takes a
single mandatory argument: a list of folders, each containing the
recordings of a same population.

```
easyAnalysis <- RHRVEasy(c("path/to/folder1", "path/to/folder2"))
```

`RHRVEasy` calculates time, frequency, and nonlinear
domain HRV indices, and then it applies hypothesis test, and corrects
the significance levels. If there are more than two experimental groups
and statistically significant differences are found, it performs a
post-hoc analysis to find out which groups have the differences.

More details about the API can be found in the package documentation
`RHRVEasy-manual.pdf` and the tutorial
`RHRVEasyTutorial.Rmd`/`RHRVEasyTutorial.R` (or
the compiled versions `RHRVEasyTutorial.pdf` and
`RHRVEasyTutorial.html`).

## Data

The folder `data` (named `RRData` in the
repository) contains a zip file with the data used to test the package
in the paper. After unzipping, these data can also be used to test the
package or follow the tutorial (see next section). The folder also
contains `paperExperiments.RDS`, a data.frame with the
results of the paper experiments (see the tutorial for more
details).

## Tutorial

The `RHRVEasyTutorial.Rmd`/`RHRVEasyTutorial.R`
provides a step-by-step introduction to the package. Furthermore, the
results of the paper can be reproduced by completing the tutorial. To
follow the tutorial, please refer to the `Data` section and
unzip the zip folder under the `RRData` directory.

Note that to use the R Markdown version of the tutorial, a LaTex distribution is
needed. We also recommend installing Rstudio. After
installing LaTex+Rstudio, open the `RHRVEasyTutorial.Rmd`
file and click on the `Knit` button to compile the tutorial.
(If a pop-up window appears, asking you to install dependencies, please
accept this prompt.)

If not LaTex distribution is installed, you may use the R script
`RHRVEasyTutorial.R` to run the tutorial.
